# Understanding Consumers to Enhance Demand for Sustainable Diets: Comment on “Adopting Sustainable Dietary Patterns: Effects of Food Labeling, Food Choices, and Eating Behaviors”

**DOI:** 10.3390/nu17010070

**Published:** 2024-12-28

**Authors:** Christopher P. F. Marinangeli

**Affiliations:** Protein Industries Canada, Centre for Regulatory Research and Innovation, 200-1965 Broad Street, Regina, SK S4P 1Y1, Canada; christopher@proteinsupercluster.ca

The creation of sustainable food systems is a “wicked problem”; it is multifaceted with no single or one-size-fits-all solution [1]. Initiatives that aim to solve specific problems related to food system sustainability can differ between geopolitical regions, countries, and communities. Increasingly, sustainability is becoming an essential component of food systems and is generally measured against four domains: human health and nutritional adequacy, ecological preservation, social equity, and economic prosperity [2,3]. Each is critical for a food system to be both sustainable and successful in its own right. The interplay between these domains cannot be overlooked, as effects on one can affect others. The complexity of food systems requires that multiple stakeholders across producers, industry, and government work to leverage resources that create efficiencies, establish systems, and develop new technologies that affect outcomes related to sustainability. However, resource allocation affects how these domains are prioritized, which can accelerate or decelerate activities that affect metrics related to sustainability outcomes [4,5]. While many attributes of food system sustainability occur between the production and distribution of food in retail, the importance of consumers cannot be forgotten [6]. They are an integral stakeholder of food systems that can create demand for sustainable solutions.

This Special Issue on “Adopting Sustainable Dietary Patterns: Effects of Food Labeling, Food Choices, and Eating Behaviors” focuses on consumer-centric factors related to the adoption of sustainable dietary patterns. The ability to find solutions that align with consumer needs and values which are related to food choices can enhance the demand and value proposition for upstream sustainability efforts that occur prior to the point of purchase [7]. This Issue covers a range of topics that highlight the challenges and the need for tailored approaches to creating inclusive food environments for consumers while aligning with global objectives for sustainable food systems. They have important implications for the adoption of sustainable diets, including 1. the examination of the sociodemographic characteristics of food environments; 2. the nutritional profiling of plant-based diets and targeted consumer needs; 3. the role of policy and regulatory modernization in changing food behaviors; and 4. the importance of consumer beliefs and value systems that affect food choices. This Editorial provides a brief synopsis of the studies and reviews included in this Special Issue.

## 1. Food Environments and Socioeconomic Factors

Consumers and their food environments can be diverse. As such, understanding food environments and the unique problems that affect consumer food choices can allow the identification and prioritization of the areas where resources are needed in order to ensure the highest impact on sustainability outcomes. Lewis et al. evaluated the post-pandemic individual and household characteristics of those who regularly used corner/convenience stores as their main sources of food in Baltimore, Maryland. Most respondents had low incomes, were Black (62.2%), and did not own a home (66.9%). These individuals had a lower intake of calories from sugar-sweetened beverages compared to White participants, but also consumed fewer servings of fruit and vegetables (3.20 vs. 4.68 servings/day). Fiber intake was also higher (18.41 g/day) amongst White compared to Black individuals (12.71 g/day). Income, home ownership, and food security status also had differing effects on beverage, fruit and vegetable, and fiber consumption. The authors argue that consumers are critical in the “supply-and-demand” feedback loop that dictates food offerings in retail environments.Understanding their needs in complex urban environments can create demand and access to healthy foods. Forray et al. examined the adherence to Romanian dietary guidelines in the North-Western Regions of Romania based on sociodemographic characteristics. While most understood the link between nutrition and health, the majority of individuals (83.5%) did not adhere to the dietary recommendations, which was underpinned by a low consumption of fruit and vegetables, fish and seafood, and water intake. Interestingly, older individuals (OR = 0.98), unemployed individuals (OR = 0.40), retirees and those receiving social welfare (OR = 0.53), and those with illnesses (OR = 0.69) were the least likely to consume <5 servings of fruit and vegetables per day. It was suggested that access to affordable vegetables or the likelihood of growing their own fresh food could explain these results. The authors note the need for tailored approaches to addressing food security with linkages between food literacy and sociodemographic factors. Zhang et al. showed that, in rural China, women were the most likely to consume “traditional” (higher in vegetables/fruit and meat/seafood/eggs, and lower in milk, beans, and nuts) and “meat/animal protein” dietary patterns compared to a highly diverse “healthy” pattern. Non-obese participants had a 69% greater likelihood of consuming a “healthy” dietary pattern compared to the “meat/animal” pattern. Education level and income showed similar associations.

The results from these studies all underscore the regional specificity related to metrics of sustainable diets. They reinforce that the adoption of sustainable dietary patterns by consumers may require discrete approaches that are unique to the needs of specific food environments to affect the demand for specific dietary patterns.

## 2. Evaluating “Plant-Based” as a Sustainable Dietary Pattern

Global discussions around sustainable diets often default to the adoption of a plant-based dietary pattern. This is predicated on the simplified notions that plant-based diets and protein require fewer environmental resources to produce, can be nutrient-dense, and include higher and lower levels of fiber and saturated fat, respectively, than typical Western-type dietary patterns [8]. The fact is that a plant-based diet is an inclusive “term” that can encompass various patterns of eating, including veganism, vegetarianism (and its subcategories lacto-ovo- and pesco-vegetarian), and flexitarianism. The integration of healthy plant-based diets into dietary guidelines focuses on a high consumption of fruit and vegetables and whole grains, with the important distinction of sourcing most dietary protein from plant foods, which include legumes, nuts, seeds, and nutrient-dense processed foods such as high-protein fortified plant milks. If these patterns are integrated based on scientific evidence that support dietary guidelines, plant-based diets would be nutritionally complete and limit the consumption of nutrients of concern [9]. Innovation in the plant protein space is creating new food platforms but is heterogenous in terms of nutrient content. Studies have started to demonstrate that their use can facilitate a decrease in risk factors for cardiovascular disease [10]. Measurements of the nutritional merits of the proposed “plant-based” dietary paradigms require evaluation to ensure they align with the principles of sustainable diets.

In this Special Issue, Acosta et al. used an overall plant-based diet index (oPDI), healthful PDI (hPDI), and less healthful (lhPDI) to evaluate the nutrient and food group intakes of Canadian preschool-aged children. Their results demonstrated that different indices can be used to evaluate nutrient intakes and associated food groups. For the oPDI score, both healthy and unhealthy plant foods were scored positively. Healthy plant foods were scored positively for the hPDI, while unhealthy plant foods were scored negatively. The scoring was the opposite for the lhPDI. Animal foods were scored negatively for all indices. Using the oPDI, children in the highest tertile had higher (folate, iron, fiber) and lower intakes (calcium, vitamin D, vitamin B12) of nutrients and foods to encourage. Protein was also lower given the negative scoring for animal protein, as was as saturated fat intake. The diets that generated the highest hPDI scores showed higher intakes of fiber, folate, and iron, and a lower consumption of cholesterol and saturated fat compared to Tertile 1. Lastly, the lhPDI scores in Tertile 3 showed a greater intake of added sugar and carbohydrate alongside a lower intake of protein and various vitamins and minerals, including calcium, vitamin D, vitamin B12, folate, and iron. Across all indices, fruits and refined grains were the primary plant food groups consumed, with relatively minor contributions from legumes, nuts, and seeds. These indices can be used to identify nutritional gaps in plant-based dietary patterns to be addressed. Fulgoni et al. used the National Health and Nutrition Examination Study (2013–2018) to show that nutrition adequacy for some varied across age groups. For example, the highest quartile of plant protein amongst adolescents showed a decrease in calcium, potassium, and vitamin D adequacy and an increase in adequacy for copper and magnesium. For adults 19–50 years of age, protein and vitamin B12 adequacy decreased, but folate and iron adequacy increased. Furthermore, zinc and calcium adequacy were reduced for older adults (≥51 years). These results emphasize that as plant protein is promoted to become a more prominent attribute of dietary patterns, care is needed to ensure nutrient intakes are sufficient. In order to achieve this, planning and shifts toward a greater incorporation of plant protein foods not typically prominent in typical US dietary patterns, such as legumes, nuts, and seeds, are required. Furthermore, depending on the level of animal proteins consumed in a diet, the fortification of manufactured foods could be a valuable tool for maintaining the adequate intake of some nutrients. Accurate dietary assessments and newly developed assessment tools can assist with the adoption of sustainable plant-based diets that are nutritionally adequate.

Sustainability efforts and targets can change across the lifespan, given that nutrient requirements can also change over time. Metabolic perturbations, health status, and socioeconomics can also affect nutrient needs and food accessibility. Wakayama et al. developed and validated the Meiji Nutritional Profiling System that can assess the nutritional density of innovative food products for adults ≥ 65 years in Japan. With shifts in dietary patterns being used to drive sustainability efforts, sufficient guidance and access to tools could be helpful for highlighting challenges for specific populations. This ensures that diets are nutritionally adequate by recommending specific foods and strategies that address individual needs.

## 3. Steering Consumers Toward Sustainable Food Behaviors with Regulation and Policy

The role of regulations and policies as facilitators of consumer food choices cannot be ignored. They create rules by which nutritional attributes can be communicated and can be used as tools to help address food system sustainability. Levis et al. summarized the results of a 7-day workshop organized by the Pan American Health Organization and World Health Organization in July 2022. The results generated a variety of objectives and population-focused tactics aimed at improving the nutrient density of diets and decreasing the risks of non-communicable disease. The highlighted regulatory and policy initiatives included labeling, taxation and tax incentives, marketing, school programs, and conflict-of-interest mitigation. It was also emphasized that efforts should be coordinated to create efficiencies for implementation. Onyeaka et al. systematically evaluated the global policy and regulatory initiatives that have helped address food security and its effects on mental health. Integrated approaches have been successful in addressing both conditions, but tactics are regionally specific given the different challenges and infrastructures in place across food systems. The successful case studies discussed were targeted social safety net programs and tax reductions (West and Central Africa), humanitarian efforts through the FAO, World Trade Organization, and Group of Seven countries (Europe), the Supplemental Nutrition Assistance Program (US), Universal Childcare Benefit (Canada), community food programs as a replacement for food banks (UK and Canada), and the electronic Public Distribution System used to streamline the distribution of subsidized grain (India). The authors also state that food security policies that combine social, economic, and healthcare outcomes will address food security and have concomitant benefits on mental health. Regulation and policy outcomes should be continuously evaluated to reflect the present food system and create sustainable food environments for consumers.

## 4. Driving Sustainable Food Choices by Aligning with Consumer Beliefs and Values

Personal beliefs and values around food are an important consideration when it comes to food choices. It is these personal ideals and food behaviors that can affect the sustainability of food systems. Consumer insights are a useful tool that can be leveraged for successful food innovation, research, messaging, and merchandizing, as well as the implementation of effective regulations and policies that support sustainable food purchase. Yang et al. evaluated perceptions and support for front-of-pack labeling on menus in Canada. Most respondents indicated that they would like to see more health logos that outline healthier options (27% of respondents) and foods with high amounts of nutrients of concern (21% of respondents). However, when asked whether healthy logos would affect their eating behaviors, 44% of consumers indicated that logos would not affect whether they ate at a restaurant. Furthermore, 62% indicated that they would still consider buying a menu item associated with a health warning logo. Given that “health” was the fourth most important factor given for eating outside the home (after taste, price, and convenience), health logos may not have a strong value proposition for food items in restaurants. Other tactics could be more successful for facilitating healthier food choices when eating outside the home. The authors highlight that a multitude of factors affect food choices, including socioeconomic status, and that multidisciplinary approaches are required to facilitate the purchase of healthier foods inside and outside the home.

The results of Hayashi et al. highlight that consumer food beliefs can differ within the same population demographic. An analysis of the “Survey on Dietary Habits of the Younger Generation” (n = 1888) showed that amongst 18–35-year-olds in Tokyo, Japan, three attitudes toward healthy and balanced meals emerged: 1. “Valuable yet burdensome”; 2. moderate value, but “environment-reliant”; and 3. “low value due to hassle.” Various attributes were associated with each attitude. For example, the group that viewed nutritious meals as having “low value due to hassle” was associated with having a lower income, and was more concerned with taste, price, and the effort required in preparing and consuming a balanced meal. Those who assigned the greatest value to the consumption of healthy, balanced meals had the skills and knowledge for ease of adoption. The authors emphasize that tactics aimed at improving diets should be dynamic and tailored to the knowledge, beliefs, attitudes, and socioeconomic indicators of the target group.

What drives purchase intent? Segmenting and understanding consumer food values can predict willingness to pay. Albornoz et al. investigated the effects of perceived value on the willingness to pay for healthy branded food using a sample (n = 518) of Peruvian consumers of the healthy brand “Union Brand”. The results demonstrated that being health-conscious was associated with the perceived quality, social value, emotional value, and financial value of a food. Furthermore, all of these “perceived values”, except for financial value, were associated with a willingness to purchase healthy foods. It was hypothesized that the non-association between financial value and willingness to pay was due to consumers placing higher value on quality. For consumers of healthy foods, perceived value can facilitate purchase decisions where the industry can tailor communication strategies toward successful sales. It could be worthwhile to apply the same methodology to other consumer demographics to find values to align with sustainability outcomes.

Although plant-based dietary patterns represent a fundamental construct around sustainable diets, adoption can be a challenge. For consumers identifying as vegan or vegetarian, there is little guidance for those who self-identify as flexitarian/or semi-vegetarian [11]. In alignment with some of the other results presented in this Issue, the primary drivers for food purchase decisions have consistently been taste > price > health > convenience > environment [12]. In the context of plant-based diets, there is value in understanding sustainability attributes and attitudes toward food choices.

Consavage Stanely et al. conducted a secondary analysis of the International Food Information Council’s Food & Health Survey (2012–2022) to evaluate the perception, beliefs, and behaviors related to plant-rich dietary patterns. While environmental sustainability was perceived to be positive, it influenced less than half of purchase decisions. From 2020 to 2022, the proportion of respondents that reported eating somewhat or much more red meat increased from 13.1 to 18.7%. Furthermore, >50% of older consumers were more inclined to eat the same amount of red meat vs. <45% of younger consumers. Between 2019 and 2022, adherence to plant-based dietary patterns was low, with vegan and vegetarians making up an average of 4.6% of respondents. Flexitarian patterns ranged between 2.1 and 7.4%, while “plant-based” dietary adherence ranged from 4.0 to 11.8%. These results emphasize the “say-and-do” gap between food beliefs and behaviors. Understanding how consumers can translate their food beliefs into actional behaviors in the retail environment could be a valuable strategy for driving the adoption sustainable food behavior promotion.

## 5. Final Thoughts

The 12 papers presented in this Special Issue on “Adopting Sustainable Dietary Patterns: Effects of Food Labeling, Food Choices, and Eating Behaviors” report on a variety of consumer-centric topics related to sustainable diets. Given the complexity of consumers with diverse needs, food beliefs, and values, different tactics are required to encourage their interest in embracing sustainable diets. Also, it is important to acknowledge that consumers might not be interested in the sustainability attributes of their food. However, this does not preclude strategies from promoting foods associated with better sustainability characteristics by communicating on those attributes that do matter to those consumers. Analytical tools can assess the nutritional merits of diets, while regulation and policy can expedite efforts. Sustainability is a “wicked problem” and stakeholders across the food value chain will continue to design initiatives that advance it in various domains. However, by integrating approaches that target consumers, demand, and sustainable food behaviors, the success of these efforts across the food system can be magnified.

## References

[1] Clark M., Macdiarmid J., Jones A.D., Ranganathan J., Herrero M., Fanzo J. (2020). The Role of Healthy Diets in Environmentally Sustainable Food Systems. Food Nutr. Bull..

[2] Kraak V.I., Aschemann-Witzel J. (2024). The Future of Plant-Based Diets: Aligning Healthy Marketplace Choices with Equitable, Resilient, and Sustainable Food Systems. Annu. Rev. Public Health.

[3] Drewnowski A., Finley J., Hess J.M., Ingram J., Miller G., Peters C. (2020). Toward Healthy Diets from Sustainable Food Systems. Curr. Dev. Nutr..

[4] Gustafson D.I., Decker E.A., Drewnowski A., Hamm M.W., Hwang J., Merrigan K.A. (2022). Making Healthy, Sustainable Diets Accessible and Achievable: A New Framework for Assessing the Nutrition, Environmental, and Equity Impacts of Packaged Foods. Curr. Dev. Nutr..

[5] Miller K.B., Eckberg J.O., Decker E.A., Marinangeli C.P.F. (2021). Role of Food Industry in Promoting Healthy and Sustainable Diets. Nutrients.

[6] Fanzo J., Drewnowski A., Blumberg J., Miller G., Kraemer K., Kennedy E. (2020). Nutrients, Foods, Diets, People: Promoting Healthy Eating. Curr. Dev. Nutr..

[7] Kenny T.A., Woodside J.V., Perry I.J., Harrington J.M. (2023). Consumer attitudes and behaviors toward more sustainable diets: A scoping review. Nutr. Rev..

[8] Alsaffar A.A. (2016). Sustainable diets: The interaction between food industry, nutrition, health and the environment. Food Sci. Technol. Int..

[9] Tso R., Forde C.G. (2021). Unintended Consequences: Nutritional Impact and Potential Pitfalls of Switching from Animal- to Plant-Based Foods. Nutrients.

[10] Crimarco A., Landry M.J., Carter M.M., Gardner C.D. (2022). Assessing the effects of alternative plant-based meats v. animal meats on biomarkers of inflammation: A secondary analysis of the SWAP-MEAT randomized crossover trial. J. Nutr. Sci..

[11] Rolands M.R., Hackl L.S., Bochud M., Lê K.A. (2024). Protein Adequacy, Plant Protein Proportion, and Main Plant Protein Sources Consumed Across Vegan, Vegetarian, Pescovegetarian, and Semivegetarian Diets: A Systematic Review. J. Nutr..

[12] International Food Information Council 2024 IFIC Food & Health Survey. https://foodinsight.org/wp-content/uploads/2024/06/2024-IFIC-Food-Health-Survey.pdf.

